# Experience in diagnosis and treatment of pediatric fibular osteomyelitis

**DOI:** 10.3389/fsurg.2025.1671156

**Published:** 2025-11-11

**Authors:** Yuan Liang, Zhenjiang Liu, Xin Wang, Qiang Ma, Xinyong Hu

**Affiliations:** Department of Orthopedics, Capital Center for Children's Health, Capital Medical University, Capital Institute of Pediatrics, Beijing, China

**Keywords:** fibula osteomyelitis, children, surgical intervention, vacuum sealing drainage, conservative treatment

## Abstract

**Objective:**

To summarize and analyze the clinical characteristics, diagnosis, treatment, and prognosis of pediatric fibular osteomyelitis, and to analyze related influencing factors, providing evidence for clinical management of pediatric osteomyelitis in rare locations.

**Methods:**

A retrospective analysis was conducted on the clinical data of pediatric patients with fibular osteomyelitis from January 2018 to December 2024, including demographic characteristics, clinical manifestations, laboratory and imaging examinations, microbiological results, treatment modalities, and follow-up outcomes.

**Results:**

A total of 13 patients were included (9 males, 4 females) with a median age of onset of 7.3 years. There were 11 acute cases and 2 chronic cases. The main clinical manifestations were bone pain, fever, and local abscess formation. The positive rate of microbiological culture was 76.9%, with Staphylococcus aureus being the most common pathogen (including 1 case of MRSA). Two patients responded effectively to antibiotic therapy alone, while 11 cases required surgical intervention, including fibular fenestration, debridement, segmental resection, and VSD therapy. All patients showed significant reduction in inflammatory markers after treatment. During 3–24 months of follow-up, 3 cases experienced recurrence requiring repeat surgery, with no severe disability or deformity observed.

**Conclusion:**

Pediatric fibular osteomyelitis has an insidious onset and poses diagnostic challenges. Staphylococcus aureus, particularly MRSA, represents the primary pathogen, with higher rates of surgical intervention and recurrence compared to other long bones. Early accurate diagnosis, adequate antibiotic therapy, and individualized surgical intervention are crucial for improving prognosis.

## Introduction

Osteomyelitis represents a serious orthopedic infection in the pediatric population that can lead to acute morbidity and long-term functional impairment (1). Acute hematogenous osteomyelitis (AHO) is the most common form in children, typically affecting the metaphyses of long bones such as the femur, tibia, and humerus, which is closely related to their rich but sluggish blood supply (2). Fibular osteomyelitis is extremely rare in pediatric patients, with most literature consisting of case reports or small case series. The largest systematic review published in 2023 included only 21 cases (3). The fibula has a low infection rate due to its non-weight-bearing nature, relatively poor blood supply, and unique anatomical location (3). However, when infection does occur, atypical symptoms and limited physician experience often result in delayed diagnosis, increasing the risk of complications (4). Most affected children present with fever, localized pain, swelling, and limping, which can easily be confused with other bone and joint inflammations, infections, or injuries. Laboratory studies typically show elevated inflammatory markers but lack specificity (3). Imaging studies, particularly MRI, are crucial for early diagnosis and assessment of disease extent (5). The 2021 Pediatric Infectious Diseases Society/Infectious Diseases Society of America (PIDS/IDSA) guidelines provide recommendations for the diagnosis and treatment of pediatric AHO but do not specifically address fibular involvement (6). Fibular osteomyelitis has a high rate of surgical intervention, and delayed or inappropriate treatment carries significant risks (3). Therefore, this study retrospectively analyzes our center's experience with pediatric fibular osteomyelitis to provide guidance for clinical practice.

## Method

### Study population

This retrospective study analyzed pediatric patients with fibular osteomyelitis who were admitted to the Department of Orthopedics at Capital Center for Children's Health from January 2018 to December 2024. Inclusion criteria were: age less than 18 years and fibular osteomyelitis confirmed by clinical, imaging, or microbiological evidence. Exclusion criteria included concurrent malignancy, immunodeficiency disorders, or diabetes mellitus. This study was approved by the Ethics Committee of Capital Center for Children's Health (SHERLL2024063), and informed consent was waived due to the retrospective nature of the study.

Patient baseline data were collected through the electronic medical record system, including demographic information, clinical presentation, laboratory test results, and imaging findings.

### Patient management

Diagnostic criteria: According to PIDS/IDSA guidelines, diagnosis was based on clinical signs (fever, localized pain, swelling), elevated inflammatory markers, positive microbiological cultures, and characteristic imaging findings (particularly on MRI) such as bone marrow edema, periosteal reaction, or abscess formation.

At our institution, conservative management with intravenous antibiotics alone was indicated for patients who met all of the following strict criteria: (1) acute presentation with symptom duration less than 2 weeks; (2) absence of sequestrum or bone destruction on imaging studies (radiography, CT, or MRI); (3) no evidence of soft tissue abscess formation requiring drainage; (4) stable clinical condition without signs of sepsis or severe systemic toxicity; and (5) rapid clinical and laboratory response to empirical antibiotic therapy within 48–72 h, defined as significant reduction in local pain and swelling accompanied by declining inflammatory markers (CRP, ESR, and WBC count). Upon admission, patients meeting these criteria underwent comprehensive laboratory and imaging evaluations and received empirical intravenous cephalosporin therapy. Patients who failed to meet any of these criteria, showed clinical deterioration, or demonstrated inadequate response to initial antibiotic therapy within 72 h were considered for surgical intervention.

### Surgical management

Surgical intervention was indicated based on the following criteria: (1) failure of conservative management, defined as lack of clinical improvement or progression of symptoms despite 48–72 h of adequate intravenous antibiotic therapy; (2) severe, intractable pain significantly impairing quality of life; (3) radiographic evidence of complications including intramedullary abscess, sequestrum formation, or significant cortical/bone destruction documented on MRI; (4) chronic osteomyelitis with persistent bone destruction and non-healing despite prolonged antibiotic therapy.

For most cases, cortical windowing (fenestration) was performed to access the medullary cavity. Thorough debridement of necrotic tissue and sequestra was conducted, followed by copious irrigation with hydrogen peroxide solution and normal saline to mechanically reduce bacterial load and remove inflammatory debris. Postoperative drainage tubes were routinely placed to facilitate continued drainage. Vacuum sealing drainage (VSD) was utilized when available to promote continuous drainage and wound healing. For chronic osteomyelitis cases with extensive bone destruction showing no response to prolonged conservative treatment, segmental bone resection was performed to completely remove the infected and necrotic bone segment. The extent of resection was determined intraoperatively based on bone viability and the extent of infection. The decision for surgical intervention and choice of surgical technique were made jointly by the orthopedic team based on clinical presentation, laboratory findings, imaging characteristics, and response to initial antibiotic therapy.

### Statistical analysis

All data were analyzed using SPSS 27.0 software. Normality of continuous variables was assessed using the Kolmogorov–Smirnov test or Shapiro–Wilk test. Normally distributed continuous variables were expressed as mean ± standard deviation (Mean ± SD), and between-group comparisons were performed using independent samples t-tests. Non-normally distributed continuous variables were presented as median and interquartile range [Median (P25, P75)], and between-group comparisons were conducted using the Mann–Whitney *U* test. Categorical variables were presented as frequencies and percentages [*n* (%)], and between-group comparisons were performed using the chi-square test. When expected frequencies were less than 5 for categorical variables, Fisher's exact test was applied. A *p*-value <0.05 was considered statistically significant.

## Results

During the study period from January 2018 to December 2024, we diagnosed and treated a total of 76 pediatric patients with confirmed lower extremity osteomyelitis. Among these, 13 cases (17.1%) involved the fibula, which forms the cohort of the present study. The anatomical distribution of osteomyelitis in our overall cohort was as follows: femur 22 cases (28.9%), tibia 24 cases (31.6%), fibula 13 cases (17.1%), and foot bones 17 cases (22.4%). This distribution demonstrates that fibular osteomyelitis represents a substantial proportion of lower extremity osteomyelitis in our center, ranking third after tibia and femur. As a tertiary referral center and major children's hospital specializing in complex pediatric orthopedic infections, our institution receives a considerable number of challenging and atypical cases from surrounding regions, which may contribute to the observed proportion of fibular involvement.

A total of 13 patients were included in this study, comprising 9 males and 4 females, with a median age of onset of 7.3 years (interquartile range 3, 11.5). The clinical presentation was predominantly acute (11 cases), with 2 chronic cases. Regarding microbiology, 10 patients had positive purulent cultures, with Staphylococcus aureus being the primary pathogen (including 1 case of methicillin-resistant Staphylococcus aureus), while 3 cases had negative cultures. The distribution of infection sites included 4 cases on the right side, 8 cases on the left side, and 1 bilateral case. Anatomically, 4 cases involved the proximal fibula, 5 cases the mid-shaft, and 4 cases the distal fibula. Clinically, 12 patients presented with fever, 10 developed local abscesses, and 12 experienced bone pain (Table 1).

**Table 1:** Baseline characteristics of children with fibular osteomyelitis.

Patients number	Age (years)	Sex	Hospital stay (days)	Clinical manifestations	Disease Course	Site of infection	Bacterial culture	Treatment
1	11	Male	20	Fever, Bone pain	Acute	Right proximal fibula	SA	Fibular fenestration with VSD
2	12	Male	44	Fever, Abscess, Bone pain	Chronic	Left fibular shaft	SA	Fibular segmental resection with external plaster cast immobilization
3	3	Female	42	Fever, Abscess, Bone pain	Acute	Left distal fibula	MRSA	Fibular lesion debridement with VSD
4	12	Female	27	Fever, Abscess, Bone pain	Acute	Right proximal fibula	SA	Fibular fenestration with VSD
5	3	Male	34	Fever, Abscess, Bone pain	Acute	Left distal fibula	Negative	Fibular lesion debridement with VSD
6	10	Male	20	Bone pain	Acute	Left distal fibula	SA	Intravenous anti-inflammatory therapy
7	6	Male	26	Fever, Abscess, Bone pain	Acute	Left middle-lower fibula	SA	Fibular fenestration with VSD
8	2	Female	31	Fever, Abscess, Bone pain	Chronic	Left distal fibula	SA	Fibular lesion debridement with VSD
9	7	Male	9	Fever, Abscess, Bone pain	Acute	Right distal fibula	SA	Fibular fenestration with drainage tube placement
10	6	Male	7	Fever	Acute	Left middle-lower fibula	SA	Intravenous anti-inflammatory therapy
11	13	Female	27	Fever, Abscess, Bone pain	Acute	Bilateral tibia and fibula	SA	Fibular lesion debridement with VSD
12	10	Male	9	Fever, Abscess, Bone pain	Acute	Left distal fibula	Negative	Fibular fenestration with drainage tube placement
13	3	Male	9	Fever, Abscess, Bone pain	Acute	Right distal fibula	SA	Fibular fenestration with drainage tube placement

SA, staphylococcus aureus; MRSA, Methicillin-resistant Staphylococcus aureus; VSD, vacuum sealing drainage.

Among the 2 chronic osteomyelitis cases, the first case was a 12-year-old male who initially presented with left calf pain following trauma approximately 6 months prior to admission to our institution. The initial presentation included progressive swelling and fever (maximum temperature 40 °C), with abscess formation on the lateral aspect of the left lower leg. He underwent three incision and drainage procedures at a local hospital, where distal fibular bone destruction was identified. Subsequently, he received debridement and vacuum sealing drainage (VSD) therapy at another tertiary hospital, with wound closure after apparent improvement. Two months before admission to our center, a soybean-sized abscess recurred at the previous surgical site with purulent drainage producing light yellow fluid. Radiographic examination revealed severe distal fibular bone destruction. At presentation to our institution, physical examination showed an old surgical scar on the lateral distal left lower leg with a small skin ulceration with granulation tissue and minimal yellow discharge. Left fibular chronic osteomyelitis with pathological fracture was confirmed by imaging studies. The patient underwent staged fibular resection with external plaster cast immobilization at our center (hospital stay 44 days). The second case was a 2-year-old female who had sustained a minor finger injury with local skin breakdown approximately 10 days before her initial hospitalization at another institution, which was initially untreated. Her condition deteriorated rapidly, developing septic shock requiring intensive care unit admission with bilateral pneumonia, right wrist and hand soft tissue infection, and multi-site osteoarticular infections. Blood cultures yielded Staphylococcus aureus. She underwent multiple surgical procedures during her initial hospitalization including left knee joint aspiration, right hand incision and decompression, left lower limb debridement, tibial osteomyelitis fenestration decompression, ankle joint debridement, and distal tibial trans-physeal pathological fracture internal fixation with Kirschner wire and plaster cast immobilization. Despite these interventions, left lower leg infection recurred and she underwent left lower limb infected tissue debridement with VSD therapy. The patient was subsequently transferred to our institution where she was diagnosed with chronic distal fibular osteomyelitis. She underwent fibular lesion debridement with VSD therapy (hospital stay 31 days) and was discharged with improvement.

**Figure 1:**
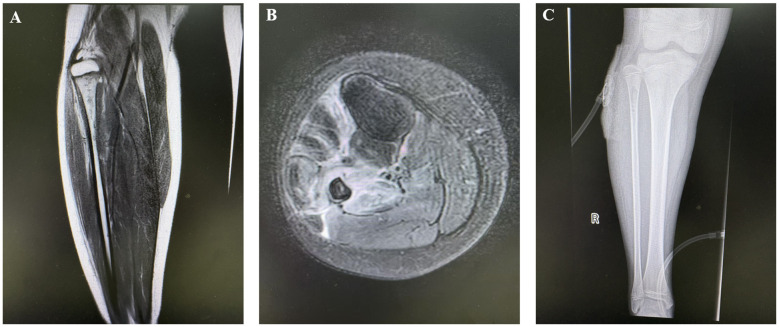
Preoperative and postoperative imaging of the patient. **(A,B)** Thickening of the periosteum in the proximal right fibula with high signal intensity. Osteomyelitis and possible bone destruction of the proximal right fibula are considered; **(C)** Irregular low-density shadow within the medullary cavity of the proximal right fibula and retained drainage tube shadow are visible.

Radiographic findings primarily showed abnormal bone density, discontinuity, and periosteal reaction in the mid-distal or proximal fibular segments, with some cases demonstrating linear low-density shadows or scattered circular lucent areas. CT examination typically revealed bone destruction in the proximal or distal fibula, irregular low-density shadows within the medullary cavity, and surrounding soft tissue swelling, with pathological fractures present in some cases. MRI findings were more sensitive, mainly showing abnormal signal intensity in the medullary cavity of the mid-distal or proximal fibula, bone destruction, periosteal reaction, and surrounding soft tissue inflammation, with some cases presenting concurrent abscess formation or involvement of adjacent joints or bones. A few patients had concurrent involvement of the tibia and talus, with imaging revealing multi-site osteomyelitis and extensive soft tissue inflammatory changes (Figure 1).

Two patients responded effectively to antibiotic therapy, while the remaining 11 patients underwent surgical intervention. Among these, 4 cases received fibular fenestration with VSD therapy, 3 cases underwent fibular debridement with VSD therapy, 3 cases had fibular fenestration with drainage tube placement, and 1 case required fibular segmental resection with external cast fixation. All patients showed improvement after treatment, with significant decreases in white blood cell count (13.3 × 10⁹/L vs. 6.5 × 10⁹/L, *p* = 0.006) and C-reactive protein levels (92.1 mg/L vs. 1.7 mg/L, *p* = 0.005) at discharge compared to admission (Table 2).

**Table 2:** Changes in inflammatory markers before and after treatment.

Parameter	Before	After	*p*
WBC (10^9^/L)	13.3 (8.7, 17.4)	6.5 (4.7, 8.8)	0.006
CRP (mg/L)	92.1 (2.7, 114)	1.7 (0.6, 5.8)	0.005

WBC, White Blood cell; CRP, C-reactive protein.

All children were followed for 3–24 months. Three patients experienced disease relapse 1–3 weeks postoperatively in our cohort, including 2 chronic cases that relapsed and 1 acute case that subsequently relapsed. 1 case undergoing debridement and suturing, and 2 cases receiving fibular segmental resection with VSD therapy. At index presentation, all 3 cases had positive bacterial cultures with Staphylococcus aureus isolated. At relapse, the 2 chronic cases had positive cultures for Staphylococcus aureus, while the 1 acute case that subsequently relapsed had negative culture at relapse. One of the 2 chronic relapsed cases presented with concurrent right hand dorsal soft tissue infection; however, this soft tissue infection showed no radiographic or MRI evidence of bone involvement, indicating isolated soft tissue pathology rather than multifocal osteomyelitis. All 3 relapsed cases had isolated fibular involvement without additional skeletal lesions, excluding the diagnosis of chronic recurrent multifocal osteomyelitis (CRMO). The first recurrent case was a 12-year-old male with chronic osteomyelitis and 6 months of fibular bone destruction. After initial staged fibular resection and plaster immobilization with 1 month of postoperative intravenous antibiotics, the wound healed well and the patient was discharged. However, one week after discharge, granulation tissue with minimal discharge appeared at the mid-portion of the surgical wound. Re-admission revealed persistent infection, and the patient underwent a second procedure involving resection of approximately 6 cm of necrotic fibula with soft tissue debridement, followed by 1 month of intravenous antibiotics, achieving satisfactory recovery. The second recurrent case was a 2-year-old female who underwent initial soft tissue incision and drainage plus fibular drilling and drainage performed one week after symptom onset. The patient was discharged with good wound healing. However, 12 days after discharge, accidental weight-bearing on the affected limb occurred, and 10 days later, exposed fibula was noted at the wound site. Re-admission imaging confirmed chronic fibular osteomyelitis with pathological fracture. The patient underwent infected tissue debridement, necrotic bone removal, and VSD therapy. Intraoperatively, extensive fibular necrosis was encountered necessitating resection of the majority of the fibular shaft, preserving only the distal physis and epiphysis (approximately 1.5 cm). Recovery after the second surgery was satisfactory. The third recurrent case was a 6-year-old male who underwent initial soft tissue incision and drainage plus tibial fenestration performed 4 days after ankle sprain with secondary infection. Wound healing was initially good. However, one month after the first surgery, minimal wound drainage recurred. Re-admission revealed persistent necrotic tissue, and the patient underwent subcutaneous and periosteal necrotic tissue debridement with wound closure, followed by intravenous antibiotics, achieving good recovery. All three recurrent cases underwent complete debridement of necrotic tissue during their second surgeries and subsequently achieved satisfactory outcomes without further recurrence during follow-up. The remaining 10 patients showed clinical improvement. At the latest follow-up, interim orthopedic outcomes were assessed. No clinically or radiographically detectable limb length discrepancy was observed in any patient. All patients were pain-free at rest and during activity after recovery. Ankle stability was clinically good in all cases, with no subjective complaints of instability. Gait was normal without limping in all patients except during the acute recurrence period. Regarding fibular-specific complications, one patient who experienced recurrence and required repeat surgery developed fibular nonunion. This patient subsequently underwent second-stage fibular resection with VSD therapy and achieved satisfactory recovery. However, given the relatively short follow-up period (median 12 months), these findings should be interpreted with caution, as growth-related deformities and alignment changes may not yet be apparent.

## Discussion

Fibular osteomyelitis has an extremely low incidence among pediatric bone and joint infections, the fibula is a non-weight-bearing bone with a relatively poorer blood supply compared to the tibia and femur, theoretically resulting in a lower risk of infection (7). However, the fibular shaft remains susceptible to direct or hematogenous spread. In our cohort, the median age was 7.3 years with males comprising 69.2%, which is consistent with other reports (3). Acute hematogenous infection represents the most common type, although direct inoculation from trauma and contiguous spread from adjacent infections can also occur. In clinical practice, acute cases typically present with lateral leg erythema, swelling, tenderness, and fever, with rapid progression. If not effectively controlled in the early stages, these cases can easily progress to chronic osteomyelitis. Chronic cases are characterized primarily by sinus tracts, sequestra, and pathological fractures. Local tissue fibrosis and compromised blood supply impede antibiotic penetration, exacerbating infection persistence and recurrence risk (3). The 2 chronic cases in our series were closely related to inadequate early diagnosis and treatment or pathogen resistance, highlighting the need for heightened awareness of disease progression and chronicity potential in pediatric fibular osteomyelitis.

Staphylococcus aureus is the primary pathogen in pediatric fibular osteomyelitis, and our study found a positive rate of 76.9%, which is higher than some reported literature rates of 50%–70% (3). This may be attributed to our more aggressive strategy for collecting purulent and tissue specimens for microbiological testing, the small sample size, and regional variations in prevalent strains. Additionally, some patients received antibiotic treatment in the early stage of disease, which may have suppressed the detection of non-Staphylococcus aureus pathogens, thereby relatively increasing the detection proportion of Staphylococcus aureus. The proportion of MRSA infections in pediatric osteomyelitis has increased significantly in recent years. Children with MRSA infections commonly present with fever, persistently elevated inflammatory markers, prolonged disease course, increased surgical requirements, and higher recurrence rates (8). One MRSA-infected patient in our series underwent two left leg soft tissue incision and drainage procedures and left fibular drilling drainage, after which the condition improved and the patient was discharged. However, recurrence occurred 2 months postoperatively requiring readmission, and the case progressed to chronic fibular osteomyelitis with extensive necrosis of the left fibula and pathological fracture. This patient ultimately required extensive fibular resection with left leg debridement and two VSD procedures, illustrating the treatment challenges and prognostic difficulties that MRSA infections pose in pediatric fibular osteomyelitis. The negative cultures in some cases suggest that early antibiotic use, sampling techniques, and atypical pathogens (such as Salmonella, Pseudomonas, Streptococcus, etc.) may all affect diagnosis, necessitating the integration of molecular detection methods and broad-spectrum anti-infective strategies.

The clinical presentation of fibular osteomyelitis lacks specificity and can easily be confused with soft tissue infections, bone tumors, or arthritis. Typical symptoms include lateral leg erythema, swelling, tenderness, limping, and fever (3). In our cohort, 92.3% presented with fever, 76.9% had local abscesses, and 92.3% experienced bone pain, which is consistent with previous literature reports (3). Inflammatory laboratory markers were significantly elevated at admission and declined effectively with treatment, demonstrating their value for dynamic monitoring, although their specificity for early diagnosis remains limited (6). Regarding imaging, MRI serves as the gold standard, sensitively detecting bone marrow edema, periosteal reaction, and abscess formation, with superior performance compared to x-ray and CT (9). In some chronic cases or early-stage infections, x-ray findings may be subtle, leading to diagnostic delays (10). In clinical practice, MRI should be performed early in children with persistent leg pain, erythema, swelling, and elevated inflammatory markers to improve diagnostic accuracy. When necessary, specimens should be obtained from multiple sites for microbiological testing.

Treatment of pediatric fibular osteomyelitis should be based on adequate doses of sensitive antibiotics combined with individualized surgical intervention. According to international consensus guidelines such as PIDS/IDSA, the recommended total duration of antibiotic therapy is 3–4 weeks, with early oral conversion possible for acute cases, while chronic or complicated cases require extended treatment courses and intensive targeted management (6). In our series, only 2 cases responded effectively to antibiotic therapy alone, with 80% requiring surgical intervention, reflecting the limitations of infection control in the fibular region and the potentially higher surgical requirements compared to other long bones. Surgical approaches include fibular fenestration, debridement, segmental resection, and VSD therapy. VSD offers advantages including dead space elimination, continuous drainage, promotion of granulation tissue growth, and enhanced local antibiotic concentrations, significantly improving infection control and wound healing rates, leading to increasingly widespread application (11). However, the long-term efficacy of VSD, its complications, and optimal indications for use in pediatric osteomyelitis require validation through multicenter prospective studies.

For chronic or extensively necrotic cases, fibular segmental or complete resection is often unavoidable, but the impact on lower limb alignment and joint stability must be carefully considered. The proximal and distal fibula directly affect knee and ankle joint mechanics, and resection can result in ankle valgus, fibular nonunion, and knee-ankle instability (12). In recent years, Ilizarov bone transport, antibiotic-loaded bone cement, and Masquelet technique have been gradually introduced for fibular reconstruction (13–15). Some cases can be managed with limited internal fixation and absorbable bone substitute materials to improve bone healing rates and preserve lower limb function (16). However, surgical planning must avoid injury to the deep and superficial peroneal nerves, with close follow-up required until skeletal maturity to monitor for growth plate damage and deformity.

The recurrence rate in our study was 23.1%, which is higher than that of most long bone osteomyelitis cases (3%–10%) (3), primarily occurring in MRSA infections, incomplete debridement, or chronic cases. Recurrence typically manifests as persistent local erythema and swelling, sinus tract formation, sequestrum development, and even pathological fractures. Aggressive secondary surgery and VSD intervention help control infection, but some cases still require long-term multiple surgeries and rehabilitation training. Short-term follow-up showed no severe disability or deformity, but literature reports indicate that fibular resection can lead to complications such as ankle valgus and knee-ankle instability, particularly requiring attention to skeletal development and limb alignment abnormalities in children during growth and development (12). Analysis of the three recurrent cases revealed several common predisposing factors. First, all three cases had inadequate initial surgical debridement with residual necrotic bone or soft tissue, underscoring the critical importance of thorough debridement at the initial intervention. The first recurrent patient had pre-existing chronic osteomyelitis with 6 months of bone destruction before our initial intervention, suggesting that chronic cases with prolonged infection may harbor more extensive necrotic tissue than initially apparent on imaging. The second recurrent patient had premature weight-bearing only 12 days post-discharge despite being only 2 years old, indicating that inadequate postoperative immobilization and activity restriction in young pediatric patients can precipitate pathological fracture and recurrence. The third recurrent patient's recurrence one month after initial debridement suggests that in some cases, particularly those with extensive soft tissue involvement, staged procedures may be necessary rather than assuming single-stage debridement is sufficient. Second, all three recurrent cases achieved satisfactory outcomes after aggressive second-stage debridement with complete removal of all necrotic tissue, demonstrating that recurrence can be successfully managed with more radical debridement.

Therefore, standardized follow-up, functional rehabilitation, and long-term evaluation are crucial for improving prognosis and reducing complications. It is important to emphasize that while eradication of infection is a necessary initial goal, the ultimate clinical concern for pediatric orthopedic surgeons is the preservation of normal skeletal growth and lower limb function through skeletal maturity. The fibula contributes to ankle mortise stability and knee-ankle alignment, and early fibular injury or resection may lead to progressive deformities that only become apparent during the adolescent growth spurt. Our cohort's short-term orthopedic outcomes—including normal gait, absence of pain, and good ankle stability—are encouraging but provisional. Definitive assessment of fibular growth, ankle mortise integrity, tibiotalar alignment, and knee stability requires longitudinal follow-up with serial radiographic measurements (including weight-bearing anteroposterior and lateral ankle radiographs, long-leg alignment views, and growth plate assessment) until skeletal maturity.

Based on our institutional experience treating 13 cases of pediatric fibular osteomyelitis, we have developed a systematic treatment algorithm. Upon admission, all patients undergo immediate blood culture collection before antibiotic administration, followed by comprehensive laboratory evaluation including complete blood count, CRP, ESR, and PCT. Imaging studies including radiography, CT, and MRI are performed to assess the extent of bone and soft tissue involvement. Empirical intravenous cephalosporin therapy is initiated immediately after blood culture collection. Clinical response is carefully monitored over the subsequent 48–72 h, with particular attention to reduction in local signs of inflammation (erythema, swelling, warmth, and tenderness) and serial inflammatory markers. Patients demonstrating rapid clinical improvement with declining inflammatory markers and meeting the previously described strict criteria for conservative management continue antibiotic therapy alone. However, patients with inadequate clinical response within 72 h, those with imaging evidence of bone destruction or sequestrum formation, or those with soft tissue abscess requiring drainage undergo surgical intervention. Antibiotic therapy is subsequently adjusted based on blood culture results and antimicrobial susceptibility testing when available. While the general principles of pediatric osteomyelitis management apply to fibular involvement, our experience highlights several fibular-specific considerations. First, the subcutaneous location of the fibula makes clinical assessment of local inflammatory signs more reliable than in deeper bones, facilitating earlier detection and intervention. Second, the non-weight-bearing nature of the fibula allows for more aggressive surgical debridement including partial or complete fibular resection when necessary without significantly compromising lower limb function, which would not be feasible for weight-bearing bones such as the tibia or femur. Third, pathological fracture of the fibula, while concerning, generally does not require internal fixation and can be managed with external immobilization alone, simplifying postoperative care. Fourth, chronic fibular osteomyelitis cases with extensive bone destruction may benefit from staged fibular resection rather than attempting preservation, as our experience demonstrates good functional outcomes even after substantial fibular loss. However, it must be emphasized that currently there is no specific treatment protocol unique to fibular osteomyelitis beyond these anatomical considerations; the fundamental principles of adequate surgical debridement, appropriate antimicrobial therapy based on culture results, and sufficient treatment duration remain universal across all forms of pediatric osteomyelitis regardless of anatomical location.

The limitations of this study include its single-center retrospective design, small sample size, case heterogeneity, and critically, the limited follow-up duration. Our median follow-up of 12 months (range 3–24 months) does not extend to skeletal maturity, which is the clinically relevant endpoint for pediatric orthopedic conditions. Consequently, we cannot adequately assess long-term growth-related outcomes, including fibular growth disturbances, progressive ankle or knee malalignment, development of valgus or varus deformities, or late-onset joint instability. These growth-related complications may take years to manifest and would only be detectable with follow-up extending to skeletal maturity (typically 14–16 years in girls and 16–18 years in boys). Additionally, surgical choices and medication regimens were influenced by physician experience and individual patient differences. The short-term outcomes reported here—such as absence of limb length discrepancy and normal gait—should be interpreted as interim findings that may not predict final skeletal outcomes.

In conclusion, pediatric fibular osteomyelitis requires early identification, precise microbiological diagnosis, adequate antibiotic therapy, and individualized surgical intervention to achieve infection eradication. However, eradication of infection represents only the initial treatment goal. The primary long-term concern for orthopedic surgeons is the preservation of normal fibular and lower limb growth, maintenance of ankle mortise and knee-ankle alignment, and prevention of progressive deformities through skeletal maturity. Our study demonstrates satisfactory short-term infection control and functional outcomes, but the limited follow-up duration (3–24 months) precludes definitive assessment of growth-related complications. Future prospective multicenter studies must include longitudinal follow-up extending to skeletal maturity, with standardized serial radiographic assessment of fibular growth, lower limb alignment, and joint stability, to establish evidence-based treatment protocols that optimize both infection control and long-term skeletal outcomes.

## Data Availability

The original contributions presented in the study are included in the article/Supplementary Material, further inquiries can be directed to the corresponding author.
